# Rhizospheric microbial communities associated with wild and cultivated frankincense producing *Boswellia sacra* tree

**DOI:** 10.1371/journal.pone.0186939

**Published:** 2017-10-20

**Authors:** Abdul Latif Khan, Sajjad Asaf, Ahmed Al-Rawahi, In-Jung Lee, Ahmed Al-Harrasi

**Affiliations:** 1 UoN Chair of Oman’s Medicinal Plants and Marine Natural Products, University of Nizwa, Nizwa, Oman; 2 School of Applied Biosciences, Kyungpook National University, Daegu, Republic of Korea; Institute for Sustainable Plant Protection, C.N.R., ITALY

## Abstract

*Boswellia sacra*, a frankincense producing endemic tree, has been well known for its cultural, religious and economic values. However, the tree has been least explored for the associated microsymbiota in the rhizosphere. The current study elucidates the fungal and bacterial communities of the rhizospheric regions of the wild and cultivated *B*. *sacra* tree populations through next generation sequencing. The sequence analysis showed the existence of 1006±8.9 and 60.6±3.1 operational taxonomic unit (OTUs) for bacterial and fungal communities respectively. In fungal communities, five major phyla were found with significantly higher abundance of *Ascomycota* (60.3%) in wild population and *Basidiomycota* (52%) in cultivated tree rhizospheres. Among bacterial communities, 31 major phyla were found, with significant distribution of *Actinobacteria* in wild tree rhizospheres, whereas *Proteobacteria* and *Acidobacteria* were highly abundant in cultivated trees. The diversity and abundance of microbiome varied significantly depending upon soil characteristics of the three different populations. In addition, significantly higher glucosidases, cellulases and indole-3-acetic acid were found in cultivated tree’s rhizospheres as compared to wild tree populations. for these plants to survive the harsh arid-land environmental conditions. The current study is a first comprehensive work and advances our knowledge about the core fungal and bacterial microbial microbiome associated with this economically important tree.

## Introduction

Symbioses of microbial communities have been of special interest to ecologists to understand their role in plant growth and development under extreme living conditions such as desert conditions [1]. Marginalize conditions often provides new insights to elucidate the basis of survival in water deficient and sever heat stress environments. Besides, plant’s unique genetic makeup, symbiosis of native microflora plays an essential role in plant life. These microorganisms, predominantly occupying the rhizosphere, enable processes involved in nutrient transport, essential secondary metabolites secretions, and abiotic and biotic factors mitigations [2–6]. The plant in return provides a favorable niche for specific kinds of microbes to grow and reproduce whilst also sharing some of the beneficial exudates and nutrients [7].

During the association with host, fungi and bacteria produces various kinds of extracellular enzymes or exozymes, which target various macromolecules such as carbohydrates, lignin, organic phosphate, proteins, and sugar-base polymers to breakdown into transportable product throughout the cells [8]. A similar prospect has been noted for indole acetic acid (IAA), a phytohormones, which help plant root and growth development during normal and stress conditions [9]. Besides initiating the host-symbiosis process, some of these exozymes and IAA counteract plant pathogenic infections and extend abiotic stress tolerance [9]. However, such interaction has been very little known about the microbial symbionts of trees growing sub-tropical deserts, especially on *Boswellia sacra* [10].

**Fig 1:**
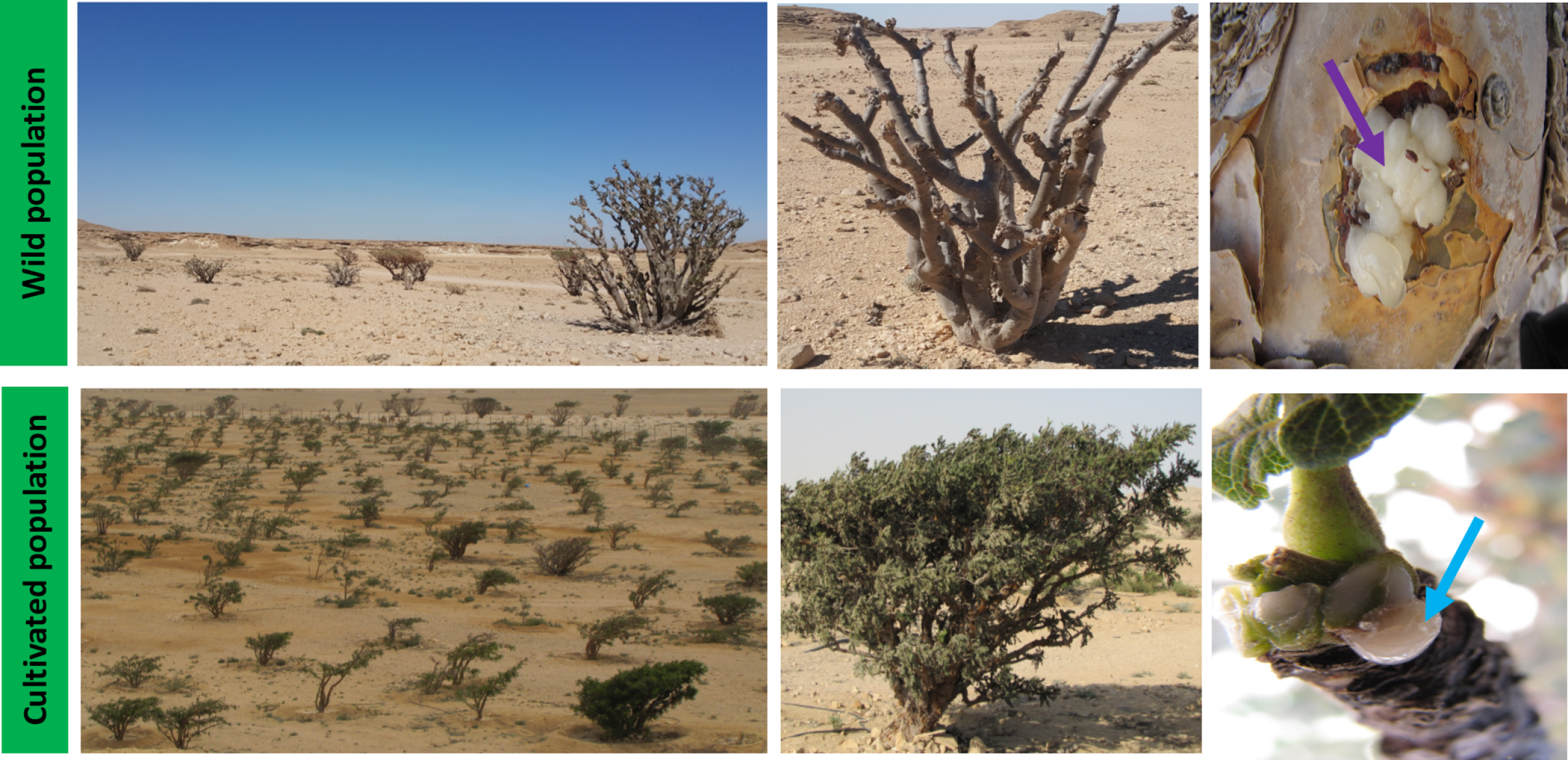
Plant habitats and their location. *B*. *scara* growing in wild and in cultivated conserved areas.

*B*. *sacra* is an economically important frankincense producing tree of the Sultanate of Oman [11]. Resin from *Boswellia* has been traded as incense from the southern coast of Arabia to the Mediterranean region for more than a millennium [12]. There are more than twenty *Boswellia* species, and *B*. *sacra* is an endemic species that grows specifically in Dhofar region of Oman. It is a keystone species that is known to provide an important oleo gum resin, which has long-standing cultural and medicinal history. The essential oil and boswellic acids have been known to possess potent anticancer activities [13, 14]. The local population obtains solid and semi-solid resin (commonly known as *Luban*) by making a series of wounds/incisions in the bark of the tree (Fig 1). The annual production of Omani frankincense ranges between 80 to 100 tons from nearly 500,000 trees [15, 16]. In some areas, the collection of resin is an economically favorable use of land than crop production and accounts for the majority of a rural household’s income [17].

Very least is known about the microbial community profile of *B*. *sacra*. Recently, El-Nagerabi et al. [18] and Khan et al. [9] have shown the endophytic microbial communities of the tree. However, looking at the tree life and its habitats, they are unique as these often experience wide array of stresses such as heat, UV incidence, drought and strong wind [1]. Moreover, there is a significant difference between the wild and cultivated trees growth, development and resin production ability. In case of economically important plants growing in such a marginalize ecosystem, very few studies have been carried out on their microflora, where some are restricted to only bacterial communities. Some of the examples includes *Rehmannia glutinosa* [19], *Rumex patientia* [20], *Polygonum cuspidatum* [21], *Aloe vera* [22] *Rhododendron arboretum* [23], Agave species [24], Cacti [25] and *Thymus zygis* [26]. In current study, we aimed to elucidate the core fungal and prokaryotic communities, and their structure in the three population of *B*. *sacra* tree through detailed metagenomics and bioinformatics approaches.

## Materials and methods

### Sampling area and collection

Rhizospheric soil samples of *Boswellia sacra* were collected from three major location at Adonab (BSA; N17°20.47’ E054°04.51), Dowkah National Park (BSD; N19°4.89’ E054°22.81) and Dowkah valley (BSW; N19°07.76’ E054°25.43) respectively in dry summer season. These locations are famous for *B*. *sacra* tree population. Rhizospheric soil samples adhering to root surface (upto 60 inches deep) were collected from these regions (Table 1). The soil analysis were performed according to Adhikari [27]. A total of fifty tree’s root regions were selected for rhizospheric soil collection from three respective sites approximately 50 km away from each other (Fig 1). The rhizospheric soil collected from each tree community was pooled into two replicates.

**Table 1:** Physical and chemical properties of soil samples collected from the rhizospheres of three *B*. *sacra* populations.

	Clay (%)	Sand (%)	Silt (%)	Bulk density (%)	Organic matter (%)	Texture	EC (dS m−1)	pH	Nitrates (mg/L)
**BSA**	13.2±0.92a	64±1.9c	17.3±0.84a	2.9±0.23a	2.6±0.39a	sandy loam	18.5±0.29a	7.82±0.43a	2.1±0.2a
**BSD**	8.2±0.12b	75.5±1.2ab	12.8±0.92ab	1.4±0.85b	2.1±0.9a	sandy loam	18.1±0.68a	6.43±0.23b	1.1±0.01b
**BSW**	4.1±0.38c	88.8±1.8a	6.1±0.43c	0.2±0.81c	0.8±0.2b	sandy	5.87±0.42b	5.49±0.1c	0.45±0.1c

Values in each column are the mean of five replications and presented with standard deviation. The different letter(s) with the values in each column showed the values are significantly different among three sampling population of *B*. *sacra*.

### Microbial products analysis in rhizospheric soil

To quantify extracellular enzymes, the method of Marx et al. [28] and Khan et al. [9] was adopted with some modifications. Briefly, all the substrates (S1 Table) were obtained from Sigma-Aldrich Co. Ltd in crystalline form. Ten milliliters of a 10 mM stock solution of each 4-methylumbelliferone (MUB) substrate was prepared, while the assay procedures were the same for each substrate. Depending on the substrate, a 7-MUB standard was used. A 10 mM stock solution of pure MUB was prepared in methanol (0.1762 g of 4-methylumbelliferone in 100 mL). This stock solution was diluted in sodium acetate (pH 5.2) buffer to 1 μM and stored at 4°C (S1 Table). The soil samples were processed for exozymes analysis using the method of Marx et al. [28] through Shimadzo (Tokyo Japan) fluorescence spectrophotometer (S1 Methods). The Indole acetic acid quantification of soil sample was performed using the method of Khan et al. [9], S1 Methods).

### Sample preparation, DNA extraction, and sequencing

The mixture of rhizospheric soil samples were multiplexed and subject to total DNA extraction through combined manual and kit based methods (Supporting Information Methods S1). PCR free libraries of each DNA sample were made by amplifying the internal transcribe spacer (3F/4R (ITS3‐4di) and 16S (V3-V4) for fungal and prokaryotic communities respectively (S1 Methods; Coleman-Derr et al. [24]. For 16S, peptide nucleic acid (PNA) clamps were used to reduce the chloroplast and mitochondrial contamination. A Paired-end 250 bp sequencing approach was performed on an Illumina MiSeq instrument (Illumina Inc., San Diego, CA, USA) operating with v2 chemistry (User Guide Part # 15027617 Rev. L). All quality sequences related to this project are available in the NCBI Sequence Read Archive (SRA) under project ID RA337739, BioProject PRJNA337739, 16S accessions (KY694695 –K694751), and ITS accessions (KY694662 –KY694694).

### Data processing and analyses

The raw Fastq reads were processed through a custom pipeline developed at the Macrogen Inc. (Seoul, South Korea; S1 Methods). Raw reads were contaminant-filtered, quality trimmed, merged and clustered to pro- taxonomic units (OTUs), respectively, at 95% and 97% identity using the UPARSE pipeline. FLASH was also used for short reads length adjustment to improve genome assemblies [29]. Taxonomies were assigned to each OTU using the RDP Naıve Bayesian Classifier [30] with custom reference databases. We used the CD-HIT-OTU package [31] and its variant tailored for Illumina reads. Another heuristic, the UCLUST greedy algorithm [32] (included in the free 64-bit version 6.0.307 of USEARCH) as implemented in the QIIME [32] script “pick_otus.py” (v1.8.0) was also used with default parameters. OTUs whose RDP classifications did not match their expected taxonomic kingdoms (Fungi and Bacteria/Archaea, respectively) were removed. Average read counts varied by sample type for different data sets. To reduce low-abundance and spurious OTUs, technical reproducibility thresholds determined empirically from technical replicates as in Lundberg et al. [33] were set and OTUs kept only if they had at least two reads in at least three samples (ITS data) or at least seven reads in at least three samples (16S data). To check for chimeric sequences amongst the different categories of sequences, the UCHIME algorithm [34] included in the free version 6.0.307 of USEARCH was used. Two variations of the program were run and compared. First the *de novo* mode in which the varying abundances of sequences in the input were exploited. Secondly, we used the reference mode in which decisions are made using a database of chimera-free sequences. Thirdly, the latest product from Robert C. Edgar titled UPARSE [35] was applied to our data (included in the free version 9.0.2132 of USEARCH). Mothur analysis were performed for community richness and diversity analysis [36].

### Statistical analysis

All samples were analyzed in triplicate. The data are presented as the mean ± standard error of the mean (SEM). Differences were evaluated using one-way analysis of variance (ANOVA). Differences were considered significant at *P* < 0.05 and were calculated by GraphPad Prism Version 6.01 (GraphPad Software, San Diego, CA, USA). The mean values were compared using Duncan’s multiple range tests at *P* < 0.05 (SAS 9.1, Cary, NC, USA). The R statistical framework version 2.11 was used for NMDS ordination plots (metaMDS), beta-dispersion (betadis), PERMANOVA (adonis), permutational ANOVA (aovp) and the estimation of diversity indices and PAST (v3.01).

## Results

### Microbial community diversity associated with the three populations of *B*. *sacra*

We assessed the prokaryotic and fungal communities of the rhizosphere of three distinctive populations of *B*. *sacra* viz. (i) BSA, (ii) BSD and their comparison with wildly grown tree population (BSW). The profiles of microbial communities were elucidated through MiSeq sequencing platform. A total of 678Mbp and 1.24Gbp of high quality read data (S2 Table) was generated for fungal and bacterial microbial communities respectively (S2 Table). The average bases counted were 1,219,536±19.1 and 546,307±14.5 for fungi and bacterial microorganisms respectively (S2 Table). The mean GC content for ITS was 49.44%; whereas it was 58.32% for 16S. The mean read count of fungal communities was 127±2.1, 995±43.2 (BSA), 98,088±5.1 (BSD) and 75,979±103.9 (BSW), whereas, for bacterial communities, it was 146,650±21.5 (BSA), 199,516±34.9 (BSD) and 188,036±44.9 (BSW). This suggests that the wild population of *B*. *sacra* tree showed a lower read count than the cultivated population (S2 Table).

**Fig 2:**
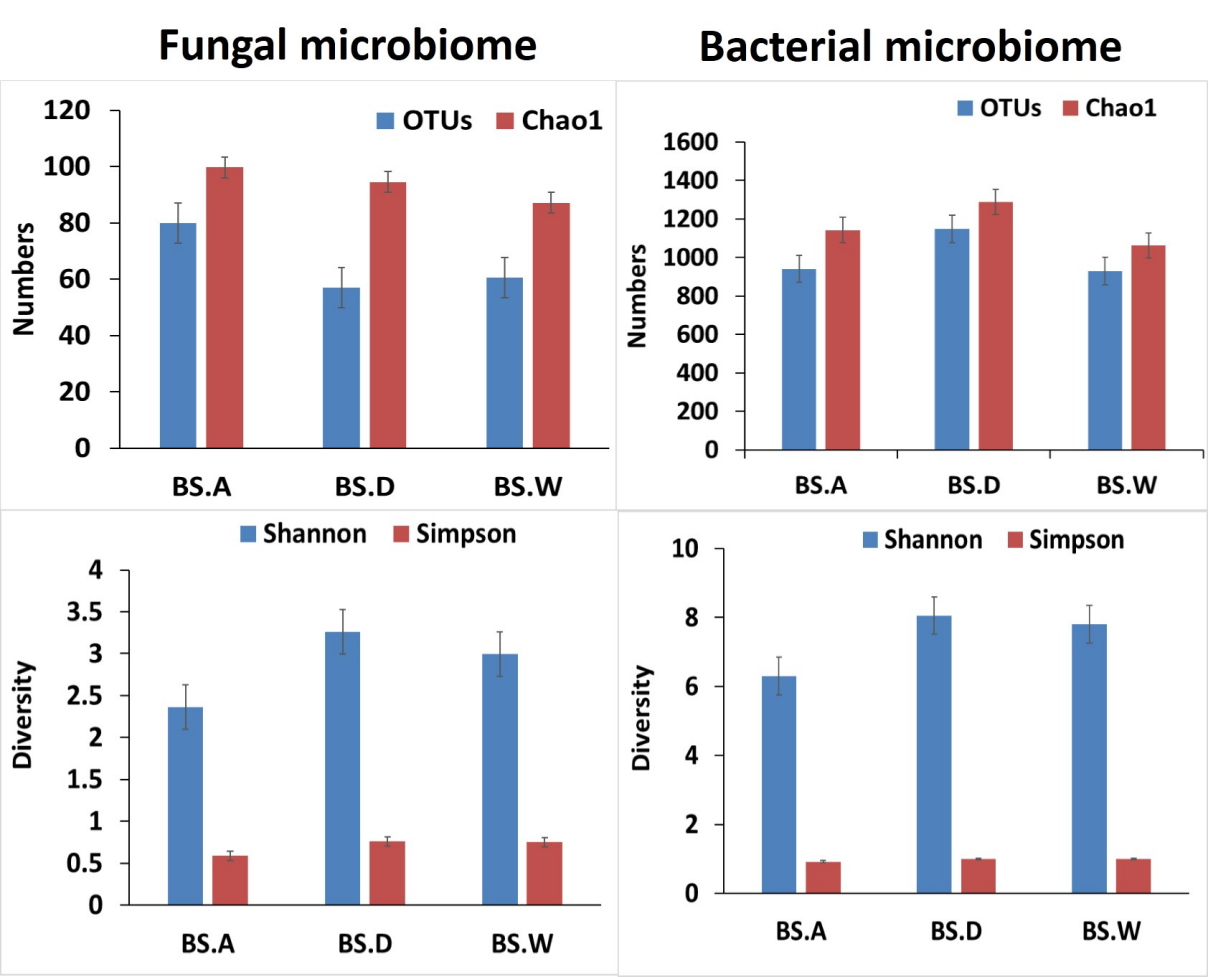
Distribution of fungal and bacterial communities operational taxonomic units (OTUs) and Chao-1 of each replica from data generated through MiSeq sequencing (16S and ITS) of the rhizospheric samples from wild and cultivated rhizosphere of *B*. *sacra* tree.

In case of OTU analysis, significantly higher (P<0.0019; average 1006±9.9) OTUs were revealed in bacterial communities, whereas, in fungi, it was significantly lower (P<0.0019; average 60.6±3.1). This suggests a higher microbial diversity of bacterial species. In case of population specific fungal OTUs, BSA showed significantly higher (*P<*0.02; 102±3.9 OTUs as compared to BSD and BSW population. However, in case of diversity indices, the Shannon was significantly higher (ANOSIM; R^2^ = 0.718, *p*< 0.00071) in BSD as compared to BSA and BSW populations of *B*. *sacra* (Fig 2). In case of bacterial communities, BSD population was found with significantly higher OTUs (*P<*0.001; 1178±13.5) as compared with other two populations. The Shannon diversity indices was also higher for BSD (Fig 2). The bacterial communities were the highest in abundance and OTUs number as compared to fungi (Fig 2). In case of nMDS of fungal communities, all the three populations and their replications were significantly distant a part from each other, however, BSW and BSD were found closely associated (R^2^ = 0.78; *P<*0.031; Fig 2).

**Fig 3:**
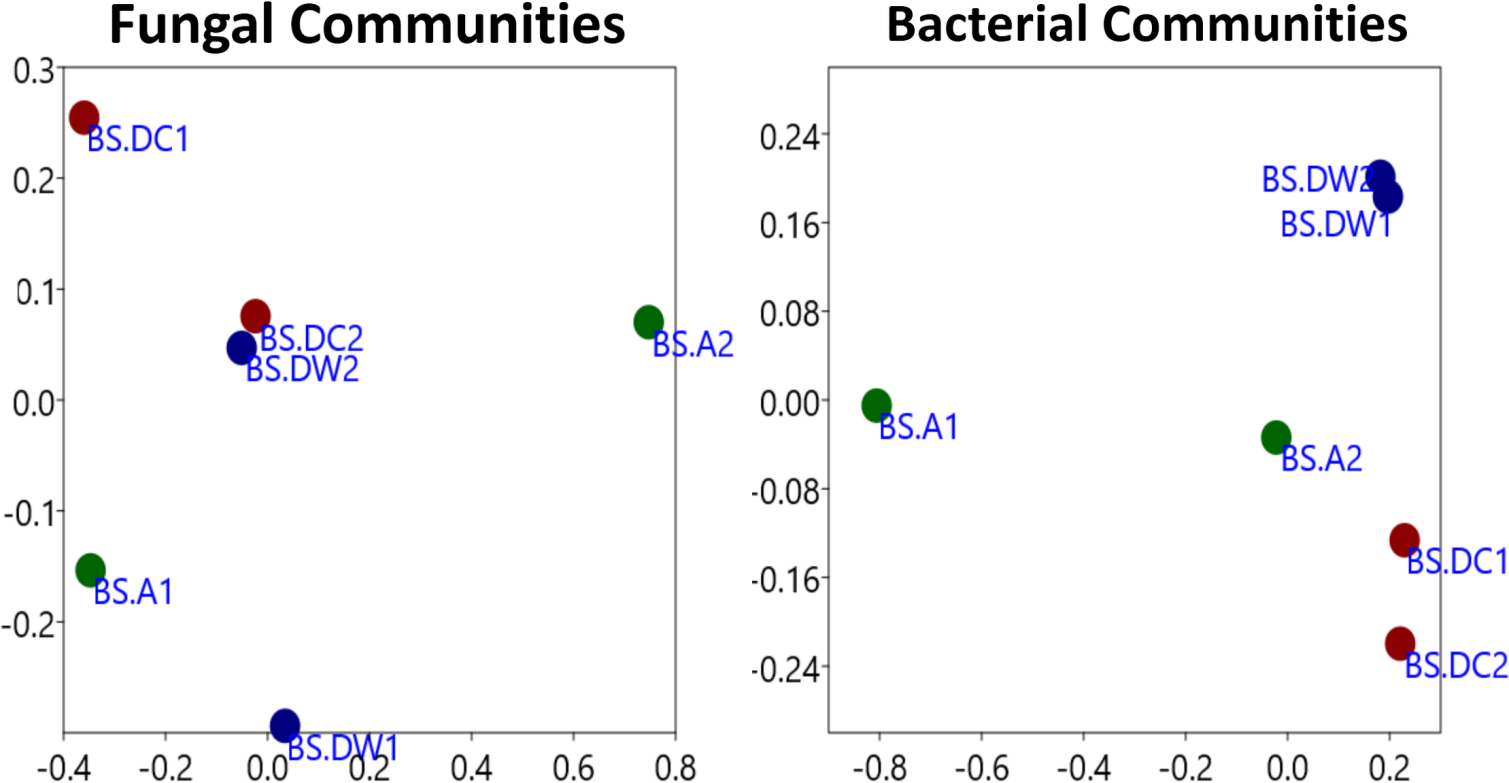
Nonmetric Multidimensional Scaling (NMDS) plots for Bray–Curtis distances of different rhizospheric populations of *B*. *sacra*. The nMDS plot was made in PAST v3.0 (New Zeeland).

In case of nMDS analysis of bacterial communities (R^2^ = 0.937; *P<*0.0092), BSW formed a separate combined group suggesting not much difference in the OTUs of the replicates. The BSA replications were significantly distant a part from each other, showing a heterogeneous diversity of fungal OTUs across same replication. The distance among replicates of BSD was not higher as compared to BSA population (Fig 3). The replicates of BSD population were comparatively not distance a part. The rarefaction curve of the three populations also revealed similar conjoining properties (S1 Fig), where fungal communities are distant apart than bacterial ones in the three samples. This was also similar to the results of hierarchal clustering (*P<*0.05 of taxa and sample), in which BSA formed a distinctive clade with BSD and BSW, instead of forming same clade with its own replication (S1 Fig). However, this was different in bacterial communities as each sample formed a cladogram with each other (S1 Fig).

### Fungal microbial communities in the Rhizosphere of *B*. *sacra*

**Fig 4:**
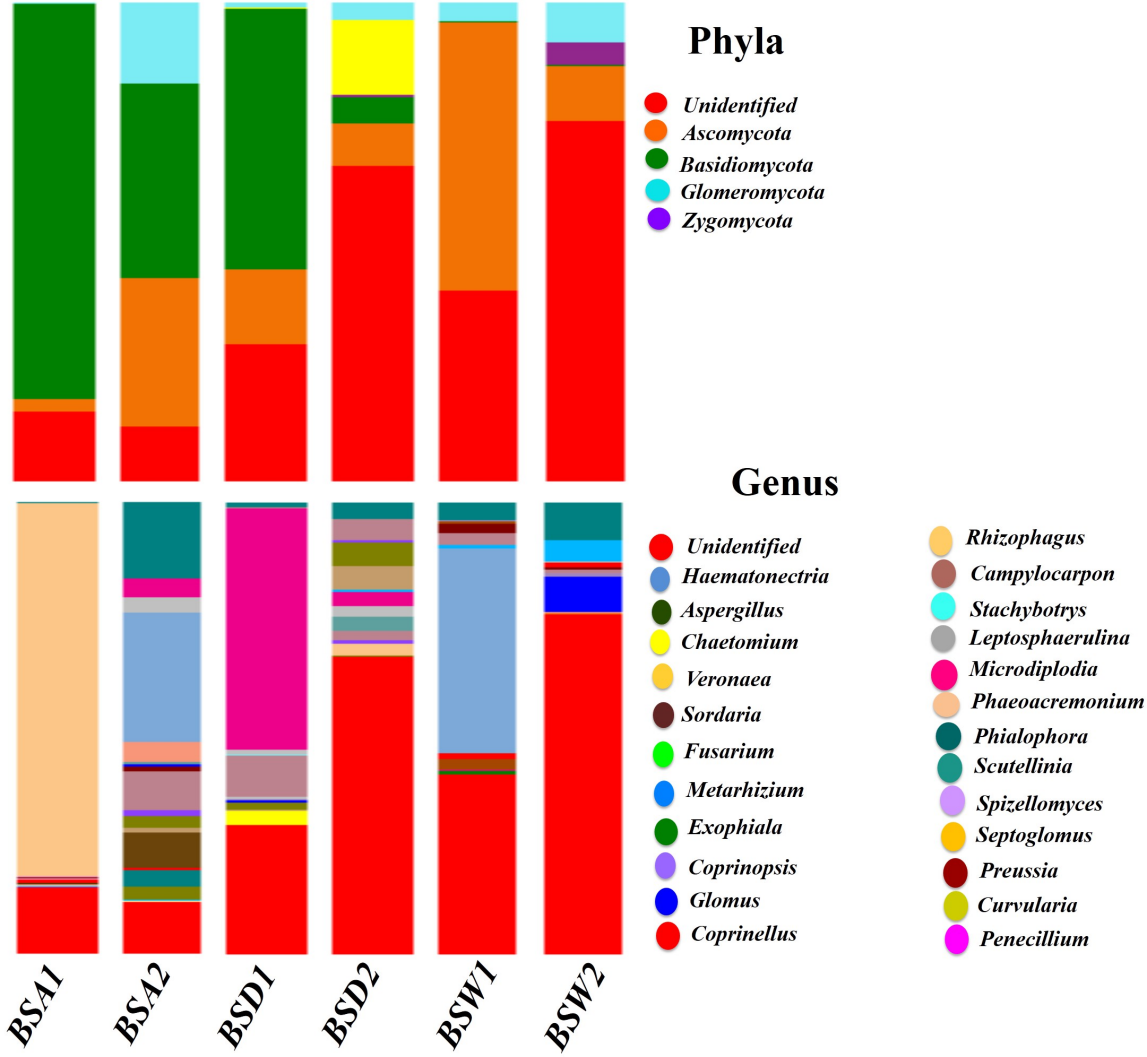
Relative abundances and shared core OTUs of fungal phyla and genus found in the rhizosphere of wild and cultivated populations of *B*. *sacra* tree. Taxa with abundance < 5% are presented as “Others/Unidentified”.

Rhizospheric microbial diversity among the three populations varied greatly. In the fungal phylum, *Ascomycota*, *Basidiomycota*, *Chytridiomycota*, *Glomeromycota* and *Zygomycota* were the abundant in BSA, BSD and BSW populations. Among populations, *Ascomycota* was significantly higher (*P<*0.001; 60%) in wild population (BSW), which was followed by BSA (47%) of *B*. *sacra*. *Basidiomycota*, on the other hand, was significantly higher (*P<*0.001; 52%) in BSA rhizosphere (Fig 4; S3 Table). The unidentified fungal communities were sharing a major proportion of data, which was about 76% in BSW and 65% in BSD populations. *Glomeromycota* and *Zygomycota* were only abundant in BSD. The diversity of fungal genus was encompassed around unidentified microbes.

In correlation with phyla, the genus too was varyingly distributed among the three populations of *B*. *sacra* (Fig 4; S3 Table). The results showed that *Haematonectria* was highly abundant (1.1 to 8.1%) in all the three populations. Where, *Aspergillus*, *Exophiala*, *Coprinopsis* and *Veronaea* were significantly higher (*P<*0.001; 4 to 7.6%) only in BSA samples as compared to BSD and BSW samples. *Glomus* and *Rhizophagus* were solely abundant in BSD population. Contrarily, *Chaetomium* and *Spizellomyces* were highly abundant (*P<*0.0001; 4 to 7.8%) in BSW population as compared to other two samples (Fig 4; S3 Table).

Among fungal species, the unidentified category showed higher proportion in the sample. The BSA, BSD and BSW population shared 45.4%, 53.1% and 62.3% respectively (S2 Fig). *Pezizaceae sp*, *Haematonectria haematococca*, and *Sebacinales sp*. were the most abundant across the three populations. Among these three species, *Pezizaceae sp* was significantly (*P<*0.00001; 22.6%) abundant in BSW population (S2 Fig) as compared to other two populations. *H*. *haematococca*, on the other hand, was 3.7, 5.2 and 1.5% in BSA, BSD and BSW populations. However, *Sebacinales sp*. *Exophiala xenobiotica*, *Veronaea botryose*, *Coprinopsis nivea*, and *Aspergillus niger* were significantly higher (*P<*0.00001; ranging from 1 to 3.8%) in OTUs abundance in BSA population only (S2 Fig).

### Bacterial diversity and the key players

**Fig 5:**
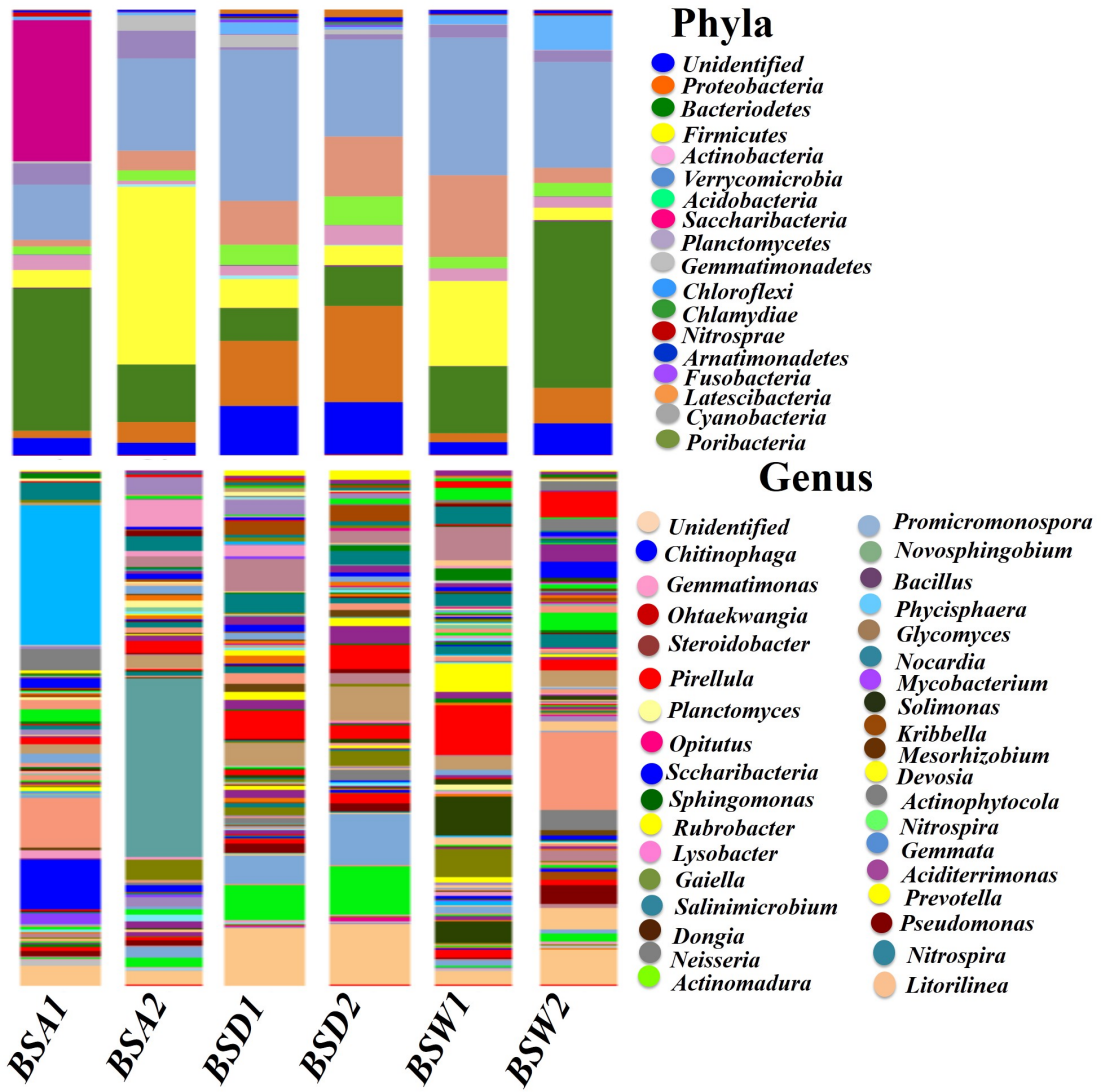
Abundances and shared core OTUs of bacterial phyla and genus from the rhizosphere of wild and cultivated populations of *B*. *sacra* tree. Taxa with abundance <5% are presented as “Others/Unidentified”.

*Acidobacteria*, *Actinobacteria*, *Bacteroidetes*, *Proteobacteria*, *Planctomycetes*, and *Gemmatimonadetes* were the most highly (*P*<0.0001) abundant bacterial phyla in the rhizosphere of the three populations. However, these varied greatly in distribution across different samples (Fig 5; S4 Table). For example, *Acidobacteria*, *Planctomycetes*. *Gemmatimonadetes* and *Proteobacteria* were significantly higher (*P*<0.0001; from 5 to 29%) in BSD rhizosphere, whereas, *Actinobacteria* was significantly higher in BSW (*P*<0.0001; 27%). *Bacteroidetes* and *Verrucomicrobia* were significantly higher (*P*<0.01; from 4 to 28%) in BSA. *Firmicutes* was significantly higher (*P*<0.0032; 5.2%) in BSW as compared to other populations. *Chlamydiae*, *Armatimonadetes*, *Chloroflexi*, *Candidatus*, *Latescibacteria*, and *Nitrospirae* were not significantly different among three populations (Fig 5; S4 Table).

The abundances of genus among different population also varied greatly as around 191 different genera were observed throughout the data. For example, *Chitinophaga*, *Steroidobacter*, *Opitutus*, *Ohtaekwangia*, *Streptomyces*, *Promicromonospora*, *Glycomyces*, and *Novosphingobium* were significantly higher (*P*<0.0001; from 1.2 to 20.1%) in BSA rhizospheric samples as compared to BSD and BSW (Fig 5; S4 Table). *Gemmatimonas*, *Pirellula*, and *Lysobacter* were significantly abundant (*P*<0.0001; from 1 to 6%) in BSD rhizospheric samples as compared to BSA and BSW. *Phenylobacterium*, *Planctomyces*, and *Sphingomonas* (*P*<0.008; from 1 to 8.6%) were significantly abundant in BSW rhizosphere as compared to BSA and BSD samples (Fig 5; S4 Table). Whereas, *Gemmatimonas*, *Ohtaekwangia*, *Gaiella*, *Mycobacterium*, and *Dongia* were commonly distributed across the three rhizospheric samples from BSA, BSD and BSW (Fig 5; S4 Table). In case of bacterial species, the abundance of uncultured bacteria was high across all the three samples. However, *Gamma-proteobacterium*, *Acidobacteria sp*., *Planctomycete sp*., *Firmicutes sp*., *Actinomycete sp*., *Escherichia coli*, *Prevotella nanceiensis*, *Chloroflexi sp*., *Rubrobacter sp*., *Lactobacillus reuteri*, *Bacillus niabensis*, and *Actinoplanes sp*. were identified and distributed across the rhizosphere of BSA, BSD and BSW ranging from 0.5 to 6.28%.

### Soil, exozymes and IAA analysis of the rhizosphere of three populations

The wild population are growing in extreme water deficiency–a desert environment, whereas, the cultivated are growing the same climate but facilitated with required water. The soil structure in these three regions (BSA, BSD and BSW) varied with respect to soil texture, pH, organic matter and nutrient concentration (Table 1), which may act as potential selection factors affecting rhizosphere communities and plant growth. Additionally, the physical structure of these three soils varied with BSA soil having a higher clay percentage (13.2%), as compared to BSD and BSW. Similarly, the highest sand percentage (88.8%) was found in BSW soil (Table 1). The Pearson’s correlation coefficients between the soil samples suggest that significantly higher (*P<0*.*05*) correlation exist for bulk density with clay, pH and nitrates; where the pH significantly (*P<0*.*001*) varies with nitrates content in the three soil samples (S5 Table).

**Fig 6:**
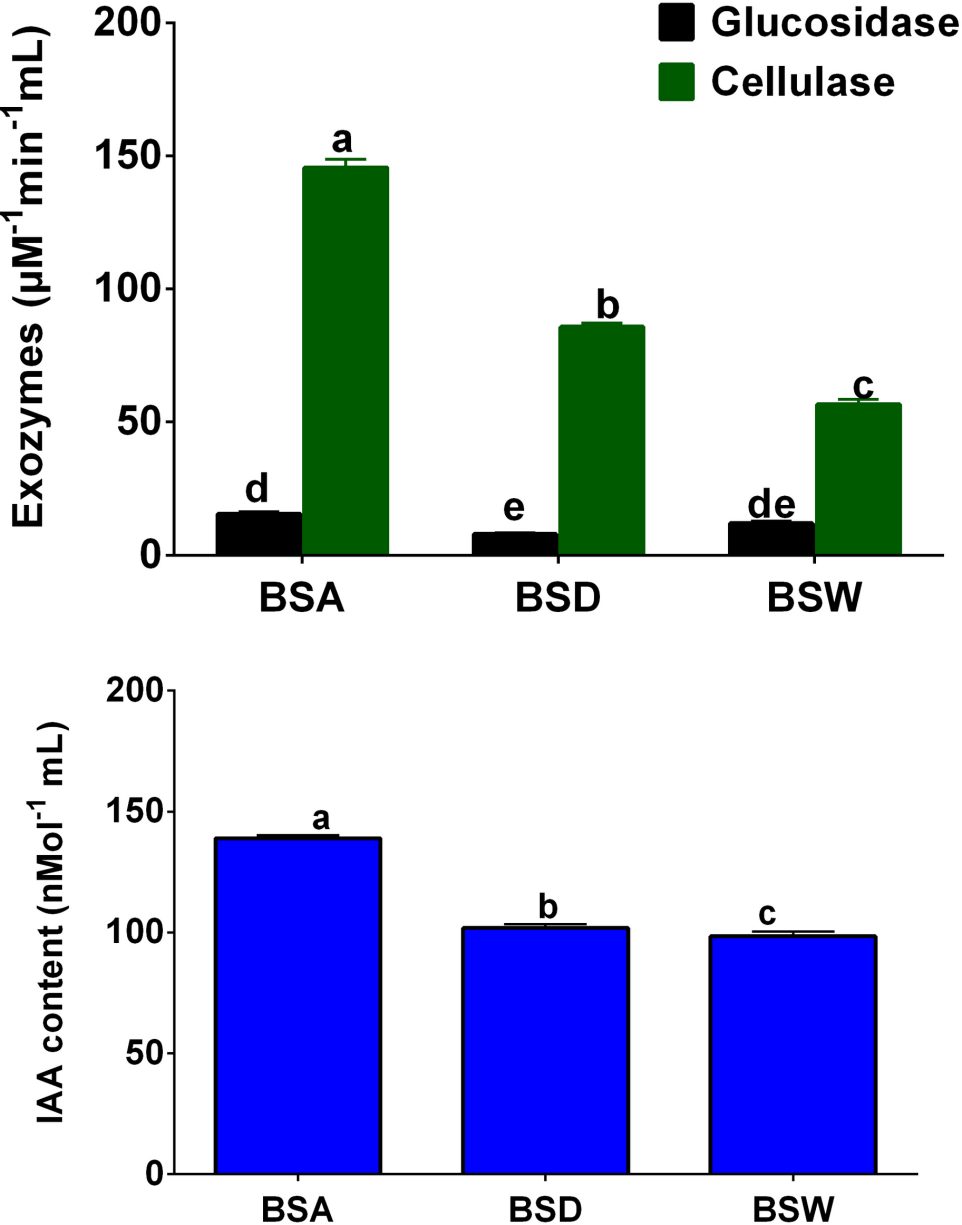
Exozymes (glucosidases and cellulases) and indole acetic acid quantification in rhizosphere of wild and cultivated populations of *B*. *sacra* tree.

The glucosidases and cellulases were quantified in the rhizospheric samples of BSA, BSD and BSW populations. The results showed that cellulases was significantly higher (*P*<0.0001; 148.4±1.8μM/min/mL) in BSA rhizospheric samples, followed by BSD samples (91.8±2.3μM/min/mL; Fig 6). In the rhizosphere of wild *B*. *sacra* population, very significantly lower amount of cellulases was found. The glucosidases were found in very low concentrations in all the rhizospheric samples, however, BSA had significantly higher (*P*<0.018; 17.1±0.9μM/min/mL) amount as compared to other two populations. In case of IAA content in the rhizospheric samples of the three populations, a significantly higher amount was detected in BSA (*P*<0.001; 137.8±2.1nM/mL) samples as compared to other populations (Fig 6).

## Discussion

The current study investigated the microbiomes associated with the rhizosphere of three populations of *Boswellia sacra* tree. The tree is endemic to Oman and possess rich affiliation culturally and economically [9]. However, its various populations both cultivated and wild are frequently exposed to regenerative threats [37]. In this regard, rhizospheric microbial communities and their diversity has been shown to be retrospective of the host plant health, growth and development [38, 39]. Results showed varying distribution of microorganisms in the rhizosphere of the three populations. Many of the current insights into the interactions and processes of rhizospheric microbiome have emerged from studies on agricultural or horticultural crop plants and model species such as *Arabidopsis thaliana* and *Medicago truncatula* [40, 41]. However, a considerable progress is also being made in understanding the microbial ecology of the rhizosphere of non-cultivated plant species in natural ecosystems [42, 43] and how microorganisms influence resource allocation, biodiversity and above-ground interactions with herbivores and their natural enemies [44, 45].

Current study elucidated the fungal and bacterial microbial communities of three *B*. *sacra* tree populations, which showed a varying response in the metagenomic data output as well as the number of OTUs. This was also demonstrated in recent studies that host-specific traits, including broad morphological characteristics and specific genetic pathways and gene products [39, 44, 45], can have significant effects on microbiome composition and diversity [24, 38, 43]. Though the climatic and soil conditions are quite similar, however, the cultivated populations (BSA and BSD) are different due to regular supply of water as compared to BSW. The cultivated and non-cultivated plants and their rhizosphere have shown to possess a varying niche of symbionts. This was previously reported by Coleman-Derr et al. [24], who showed a significant difference among different populations of *Agave* plant, which share some similarity in growth conditions with *B*. *sacra*. In conformity, Peiffer et al. [38] also showed that the structure and diversity of bacterial communities vary significantly across the rhizosphere of the same plant species collected from different location. Though, we collected rhizospheric samples from the tree with the same age and height, however, in addition to varying conditions of soil, the plant itself can influence the distribution of microbiome through different phases of development. A similar conclusion was also drawn by Chaparro et al., [40], where the bacterial community diversity changed with the seedling, vegetative, bolting and flowering stages of model plant *Arabidopsis*. [46] also showed a similar conclusion from analyzing the rhizospheric microbiota of *Solanum tuberosum* growing in different climatic conditions. [47] also showed that the location and age of the host plant can drastically impact bacterial community abundances.

Our results showed that OTUs composition varied greatly among representative replica as well as the different rhizospheric units. This too can be co-related with the plant exudation, which in turn depends on the environmental conditions where it grows. In current case, since, the rhizosphere of wild population was much deprived from the essential growth resources, therefore, it was showing lesser abundance on OTUs. Climate on the other hand, implicates the abundance of different microbial communities, thus effecting on the total OTUs of the rhizosphere. Peiffer et al. [46] also reported that different climatic results in the variation of OTUs across potato rhizosphere. A similar conclusion was also drawn for the rhizosphere of Maize and Soybean plants [48]. However, such information is scarce for the wild and cultivated economical important trees. Though, there are a few studies on the tree rhizosphere and its microbiome such as *Taxus* [41], *Quercus robur* [47], *Avicennia marina [49]* and *Populus* [50, 51]]. However, current study elucidates the microbiomes of this economically important tree for the first time. In addition, most of the studies are restricted to bacterial communities, whereas, less emphasis has been made on the fungal communities, suggesting a future need to consider the holistic approach to understand the microbiome in the rhizosphere.

Besides the abundance, the distribution of microbial communities also differed across the three population of rhizosphere. *Basidiomycota*, *Ascomycota*, *Glomeromycota* and *Zygomycota* were abundant fungal phyla in the rhizosphere of the three populations. However, the unidentified proportion in the population varied greatly specially across BSW, suggesting that it could be much higher than the available in currently available data at the NCBI. In addition, the highest composition of *Glomeromycota* and *Zygomycota* in BSW suggest a novel niche of the microbiome to support plant growth in such a harsh climatic and nutrient deficient environment. The presence of these two phyla suggest greater deterrence against pathogenic infection to the host as was shown for the rhizospheric fungi of *Panax notoginseng*, where *Zygomycota* was 46.2% in abundance [52]. This was also suggested by Shakya et al. [50] and Li et al. [53] where they have observed higher abundance of *Glomeromycota* and *Zygomycota* in *Populus deltoides* and *Pipper nigrum* L rhizospheres. This finding is consistent with previous studies on arid land plants [24, 25]. In current results, the *Haematonectria*, *Veronaea*, *Aspergillus* and *Coprinopsis* were significantly abundant in BSA and BSD, suggesting a better arsenal of the host plant to protect from harmful pathogenic fungal attack to the root structure. This was also shown by Xiong et al. [54], who compared the fungal community composition of black pepper plants and their ability to counteract against *Fusarium* wilt disease. BSW, on the other hand, showed the greater abundance of *Chaetomium* and *Pezizaceae* as compared to BSD and BSA population, which are very least reported from the arid land ecosystem. Previously, these were also found in the root zones of date palms [55], and Agave [6].

In case of bacterial communities, *Actinobacteria*, *Acidobacteria*, *Proteobacteria* and *Bacteroidete* were significantly abundant bacterial phyla from the three rhizospheric populations. These are some of the predominantly abundant bacterial species found in the metagenomic dataset obtained from various plants and rhizosphere [22, 45]. These have also been reported in some of the important medicinal plants such as *Agave* species [6], *Ginseng* [56], *Polygonum cuspidatum [41]*, *Thymus zygis* [26], *Rhododendron arboreum* [23], *Sapindus saponaria* [45], *Taxus baccata* and *Aloe vera* [22] etc. However, the distribution of core phyla such as *Proteobacteria* and *Actinobacteria* in BSW suggest higher potential of the host plant to tackle harsh environmental conditions. A similar conclusion was also shown by Kaplan et al. [57] and Marasco et al. [58], suggesting the higher abundance *Proteobacteria* and *Actinobacteria* in desert rhizosphere can help well. The significantly higher number of “Unidentified” sequences in bacteria could be due to (i) greater number of sequences of uncultured microbes, (ii) less sequenced microbial genomes, and/or (iii) absence of homologues sequence in NCBI [43].

Similarly, various abiotic factors, including soil depth, availability of soil nutrient, water content, temperature, aeration and soil management practices may affect the structure and activity of soil microbial community and abundances [59–62]. In current study, our results noticeably demonstrated that soil textural differences significantly affected bacterial population and that smaller size fractions (silt and clay) host higher bacterial community than larger size particles (sand). Among these soil textures we found that bacterial population varied greatly across different sample. The highest bacterial populations among different genus were significantly higher (1.2 to 20.1%) in BSA rhizospheric sample having higher percentage of clay and silt as compared to BSD and BSW (S1 Table). However, some genus like *Sphingomonas* and *Planctomycetes* were significantly abundant in BSW rhizosphere having highest percentage of sand (88.8%) as compared to BSD and BSA. Our results agree with previous studies that soil texture is one of the most important factors contributing soil microbial population [63–68]. Similarly, various studies reported that finer particle fractions were suitable for bacterial survival because smaller size particles provide a protective habitat for microorganism through pore size exclusion of predators [64, 69, 70]. Furthermore, higher bacterial diversity in clay and silty soil may be due to higher water contents, organic matter and nutrient availability [71, 72]. Differences in fungal community structure were observed in these samples too. Previously researchers reported effects of soil texture on fungal community and their abundances [73–75]. We found in our results that fungal communities were positively correlated with the soil sand contents and negatively with silt and clay. Similar results were reported by Wubet et al. [76] that fungal community and diversity were positively correlated to the first NMDS axis with the soil sand content and C:N ratio, while negative correlations were found with pH, silt, and clay content.

Besides, the distribution and diversity of specific microbiota, the ability of microbes in producing bioactive and potent substance in the rhizosphere have been considered essentially important for the host plant growth [77, 78]. In this mutualistic interaction, the production of bioactive metabolites such as phytohormones and extra-cellular enzymes etc can further pave a way for sustainable growth of the host [8]. As reported earlier that plant cell wall, predominantly composed of lignocellulose, serves as the main barrier to endophytic microbes. These microbes’ secrets numerous cell wall degrading enzymes, such as cellulase to break the plant cell wall to enter the plant [79]. Similarly Solomon and Matthews [80], demonstrated that the colonization of endophytes in plant internal tissues involved the production of cellulases and glucosidases [81, 82] such indicating the cell wall degrading enzymes were most likely a key determinant for the bacteria to initially enter and colonize the plant host to promote plant growth [44]. In our study, we found significantly higher amounts of cellulases as compared to glucosidases in BSA rhizosphere than BSD and BSW. A previous study showed that the endophytic symbionts with *B*. *sacra* can increase plant growth by producing IAA and extra-cellular enzymes [9]. These extracellular enzymes such as cellulases are key to maintain high organic matter for the host plant and terrestrial carbon cycle [83]. Increased amount of cellulases in BSA followed by BSD populations, suggest an active system of maintaining soil carbon influx, which on the other hand also shows a healthy soil life support system for the host. However, since, in the BSW rhizosphere, the tree populations are often confronted with extreme growth conditions, showing a minimal microbial activity, which is also in correlation with our MiSeq data for lower abundance of both fungal and bacterial communities. Ofek-Lalzar et al. [84] and Lopez-Mondejar et al. López-Mondéjar, Zühlke [83] also suggested some similar dynamics of rhizospheric communities. This is also in conformity with IAA content in BSA population. Where, IAA production has been shown a major feature of plant growth promoting activity of both fungal and bacterial rhizospheric communities as suggested by Adriaenssens [1], Cipriano et al.[85] and Khan et al.[9].

### Conclusion

The current results provide a genomic baseline to further deepen our understanding of the complex microbe interactions with plants growing in arid land ecosystem especially with *B*. *sacra*. To a certain degree our results are in correlation with recent metagenomic data on the diversity of microbiomes associated with arid/semi-arid tree populations. These plants were studied for the first time. The identification of specific taxa particularly at genus levels can provide a new insight for future research work on the associated functions and inter-play of enriched species in rhizosphere of *Boswellia* species. We also predict that secretion of exozymes, essential metabolites and soil characteristics are the key to variation in the microbial community. Current report offer an opportunities and detailed insight of core microbiome in the rhizosphere of this economically and ecologically important frankincense producing tree species.

## Supporting information

S1 Fig Rarefaction curve and hierarchical cluster analysis of the samples and their fungal and bacterial communities from three locations of *B*. *sacra* populations. (DOCX)

S2 Fig Rhizospheric fungal species composition across three different rhizospheric soil of *B*. *sacra* tree. (DOCX)

S1 Table Flourogenic enzymes and their substrates for exozymes analysis of rhizospheric soil samples. (DOCX)

S2 Table MiSeq output summary for ITS and 16S rDNA of rhizospheric region of different populations of plants. (DOCX)

S3 Table Distribution of fungal phyla and genus in the rhizosphere of different population of plants. (XLSX)

S4 Table Distribution of bacterial phyla and genus in the rhizosphere of different population of plants. (XLSX)

S5 Table Pearson’s correlation coefficients (*r*) between soil samples collected from the rhizospheres of three *B*. *sacra* populations. (DOCX)

S1 Methods Detailed methodology adopted for DNA extraction, PCR and sequencing, diversity, exozymes and indole acetic acid analysis. (DOC)
