# Supplementary material for: Rhizospheric microbial communities associated with wild and cultivated frankincense producing Boswellia sacra tree
Source: PLoS One. 2017 Oct 20;12(10):e0186939. doi: 10.1371/journal.pone.0186939 (PMC5650177; doi:10.1371/journal.pone.0186939)
Supplement: S2 Fig — (DOCX) [file pone.0186939.s002.docx]

**S2 Fig** Rhizospheric fungal species composition across three different rhizospheric soil of *B. sacra* tree

**
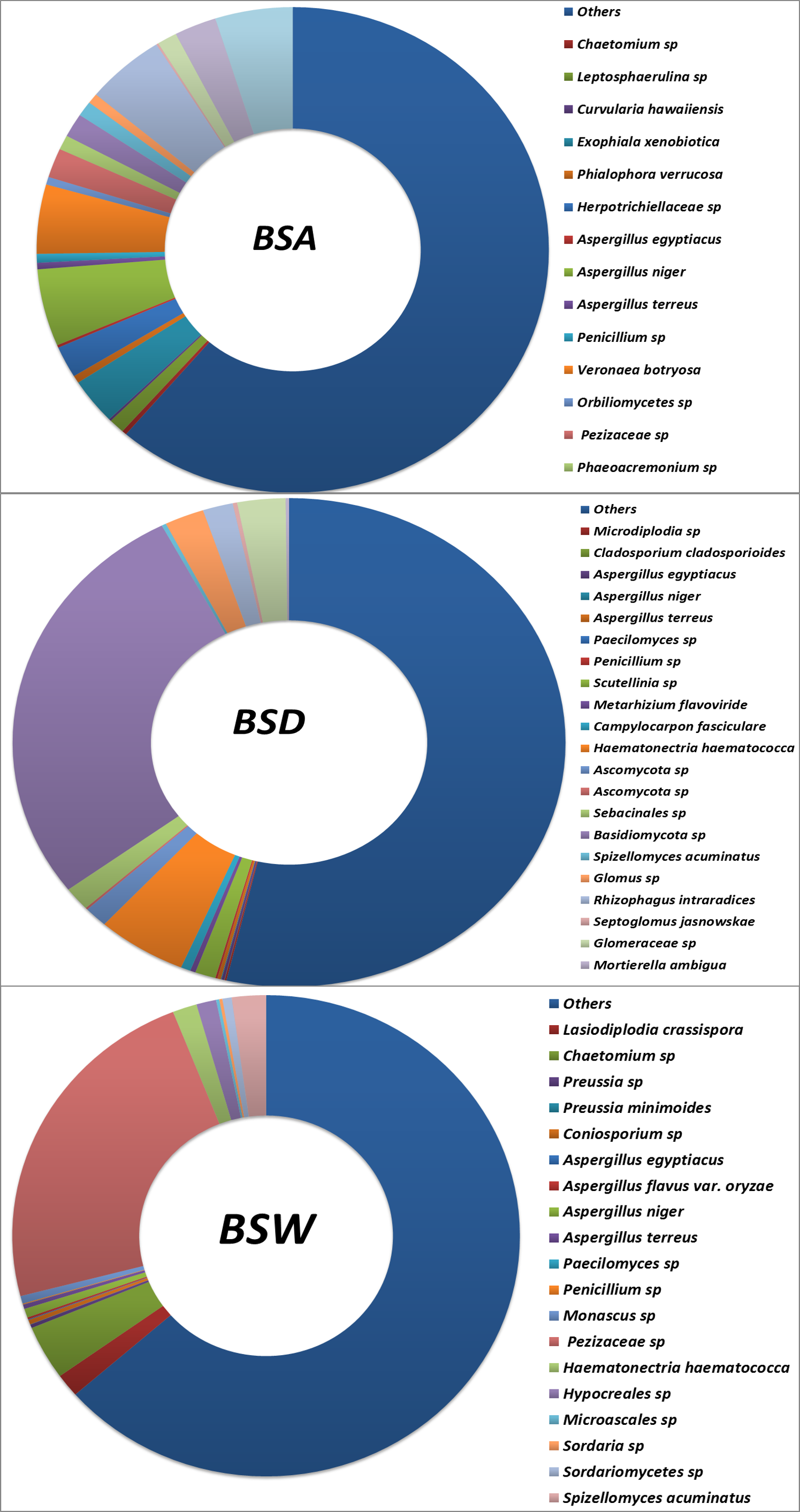
**
