# Supplementary material for: Rhizospheric microbial communities associated with wild and cultivated frankincense producing Boswellia sacra tree
Source: PLoS One. 2017 Oct 20;12(10):e0186939. doi: 10.1371/journal.pone.0186939 (PMC5650177; doi:10.1371/journal.pone.0186939)
Supplement: S1 Table — (DOCX) [file pone.0186939.s003.docx]

**S1 Table:** Flourogenic enzymes and their substrates for exozymes analysis of rhizospheric soil samples

| **Enzymes** | **Function** | **Florigenic substrate** | **Concentration** |
| --- | --- | --- | --- |
| Phosphatase | Phosphomonoesters  to phosphate | 4-MUB-phosphate  3.1.3.1 | 10 - 100 μM |
| 1,4- β -cellobiosidase | Cellulose to disaccharide | 4-MUB- β -D-cellobioside  3.2.1.91 | 10 - 100 μM |
| β-1,4-glucosidase | Cellulose to glucose | 4-MUB- β -D-glucopyranoside  3.2.1.21 | 10 - 100 μM |

MUB = methylumbelliferone
