# Supplementary material for: Rhizospheric microbial communities associated with wild and cultivated frankincense producing Boswellia sacra tree
Source: PLoS One. 2017 Oct 20;12(10):e0186939. doi: 10.1371/journal.pone.0186939 (PMC5650177; doi:10.1371/journal.pone.0186939)
Supplement: S2 Table — (DOCX) [file pone.0186939.s004.docx]

| **S2 Table:** MiSeq output summary for ITS and 16S rDNA of rhizospheric region of different populations of plants. Raw data obtained from different samples of Rhizosphere of *B. sacra* through MiSeq sequencing. The table show the data after merging (by FLASH) and clustering by CD-HIT-OTU | | | | | | |
| --- | --- | --- | --- | --- | --- | --- |
| **Sample Name** | **Total Bases** | **Read Count** | **N (%)** | **GC (%)** | **Q20 (%)** | **Q30 (%)** |
| **Fungal biome** | | | | | | |
| BSA1 | 48,311,235.00 | 119,339.00 | - | 45.01 | 98.31 | 94.40 |
| BSA2 | 50,820,033.00 | 136,651.00 | - | 49.14 | 98.25 | 93.91 |
| BSD1 | 34,877,769.00 | 90,836.00 | - | 51.02 | 97.97 | 93.09 |
| BSD2 | 40,521,426.00 | 105,341.00 | - | 49.11 | 98.13 | 93.44 |
| BSW1 | 40,002,187.00 | 105,652.00 | - | 44.48 | 98.24 | 92.35 |
| BSW2 | 18,854,197.00 | 46,307.00 | - | 50.06 | 97.57 | 92.20 |
| **Bacterial biome** | | | | | | |
| BSA1 | 94,601,739.00 | 209,258.00 | 0.00 | 56.75 | 97.68 | 92.56 |
| BSA2 | 75,305,269.00 | 166,814.00 | 0.00 | 58.63 | 97.37 | 91.60 |
| BSD1 | 92,373,639.00 | 204,430.00 | 0.00 | 56.86 | 97.67 | 92.44 |
| BSD2 | 88,079,860.00 | 194,603.00 | 0.00 | 57.44 | 97.63 | 92.37 |
| BSW1 | 57,560,171.00 | 128,920.00 | - | 57.17 | 97.77 | 92.76 |
| BSW2 | 74,187,944.00 | 164,380.00 | 0.00 | 55.44 | 97.75 | 92.77 |
