# Supplementary material for: Rhizospheric microbial communities associated with wild and cultivated frankincense producing Boswellia sacra tree
Source: PLoS One. 2017 Oct 20;12(10):e0186939. doi: 10.1371/journal.pone.0186939 (PMC5650177; doi:10.1371/journal.pone.0186939)
Supplement: S5 Table — (DOCX) [file pone.0186939.s007.docx]

**S5 Table** Pearson’s correlation coefficients (𝑟) between soil samples collected from the rhizospheres of three *B. sacra* populations

| **Parameters** | **Clay (%)** | **Sand (%)** | **Silt (%)** | **Bulk density (%)** | **Organic matter (%)** | **Texture** | **EC (dS m−1)** | **pH** | **Nitrates (mg/L)** |
| --- | --- | --- | --- | --- | --- | --- | --- | --- | --- |
| Clay (%) | 1 |  |  |  |  |  |  |  |  |
| Sand (%) | -0.99511 | 1 |  |  |  |  |  |  |  |
| Silt (%) | 0.98559 | **-0.99748*** | 1 |  |  |  |  |  |  |
| Bulk density (%) | 0.99998** | -0.99439 | 0.98438 | 1 |  |  |  |  |  |
| Organic matter (%) | 0.95288 | -0.97818 | 0.99046 | 0.95072 | 1 |  |  |  |  |
| Texture | 0.83611 | -0.8862 | 0.91685 | 0.83224 | 0.96312 | 1 |  |  |  |
| EC (dS m−1) | 0.85107 | -0.89876 | 0.92762 | 0.84736 | 0.97024 | 0.99961* | 1 |  |  |
| pH | 0.99854* | -0.98833 | 0.97502 | 0.9989* | 0.93511 | 0.80528 | 0.82149 | 1 |  |
| Nitrates (mg/L) | 0.9979* | -0.98662 | 0.97256 | 0.99833* | 0.93122 | 0.79882 | 0.81527 | 0.99994** | 1 |

* = P<0.05; ** = P<0.001; The two-tailed parametric 𝑡-test was performed to investigate the significance differences in the three populations
