# Supplementary material for: Rhizospheric microbial communities associated with wild and cultivated frankincense producing Boswellia sacra tree
Source: PLoS One. 2017 Oct 20;12(10):e0186939. doi: 10.1371/journal.pone.0186939 (PMC5650177; doi:10.1371/journal.pone.0186939)
Supplement: S1 Methods — (DOC) [file pone.0186939.s008.doc]

**S1 Methods:** Detailed methodology adopted for DNA extraction, PCR and Sequencing, diversity, exozymes and indole acetic acid analysis

**DNA Extraction**

DNA extractions for bulk soil and root zone samples were done with the MoBioTM PowerSoil DNA Isolation Kit (MoBio Laboratories, Carlsbad, CA, USA) (SDS/mechanical lysis) because of its ability to remove PCR inhibitors from the soil samples. Rhizosphere samples were first thawed on ice, pelleted at 4,000 rpm for 20 minutes at 4° Celsius, and prepped using the PowerSoil kit. Pellets ranged in size from 30 to 80 mg. Leaf episphere samples were thawed on ice and prepared using the MoBioTM PowerWater DNA Isolation Kit. For root and leaf endosphere samples, 100 mg of each frozen sample was ground to a powder in liquid nitrogen with a sterile mortar and pestle and was then prepped following a CTAB genomic DNA extraction protocol. In brief, frozen ground tissue was added to 1ml CTAB buffer (100mM Tris pH8, 20mM EDTA, 1.4 M NaCl, 2% PVP 40, 2% Hexadecyltrimethylammonium bromide, 0.2% Beta mercaptoethanol), and incubated at 65° Celsius for I hour. Samples were centrifuged at 10,000g for 10 minutes at 4° Celsius, and the supernatant was added to 1mL chloroform/Isoamyl alchohol (24:1), centrifuged, and the gDNA was precipitated with isopropanol. DNA quantity was assessed with the Qubit Broad-range Assay kit (Life Technologies, Grand Island, NY, USA).

**PCR and Sequencing**

The sequencing library is prepared by random fragmentation of the DNA or cDNA sample, followed by 5' and 3' adapter ligation. Alternatively, "tagmentation" combines the fragmentation and ligation reactions into a single step that greatly increases the efficiency of the library preparation process. Adapter-ligated fragments are then PCR amplified and gel purified.

For cluster generation, the library is loaded into a flow cell where fragments are captured on a lawn of surface-bound oligos complementary to the library adapters. Each fragment is then amplified into distinct, clonal clusters through bridge amplification. When cluster generation is complete, the templates are ready for sequencing. Illumina SBS technology utilizes a proprietary reversible terminator-based method that detects single bases as they are incorporated into DNA template strands. As all 4 reversible, terminator-bound dNTPs are persent during each sequencing cycle, natural competition minimizes incorporation bias and greatly reduces raw error rates compared to other technologies. The result is highly accurate base-by-base sequencing that virtually eliminates sequence-context-specific errors, even within repetitive sequence regions and homopolymers.

The Illumina Miseq generates raw images utilizing MCS (MiSeq Control Software v2.2) for system control and base calling through integrated primary analysis software called RTA (Real Time Analysis. v1.18). The BCL (base calls) binary is converted into FASTQ utilizing illumina package MSR (Miseq Reporter).

For Fungal ITS2 amplification, we used the ITS9F (5’ - GAACGCAGCRAAIIGYGA- 3’) and ITS4R (5’ –TCCTCCGCTTATTGATATGC- 3’) primer sets. For 16S rRNA v4 amplification, we used the established primer pair 515F (5’ – GTGCCAGCMGCCGCGGTAA- 3’) and 816R (5’ GGACTACHVGGGTWTCTAAT- 3’) primer set, along with PNA clamps to reduce chloroplast and mitochondrial contamination as in (Lundberg *et al.*, 2013). The use of PNAs in the 16S v4 amplification substantially reduced overall chloroplast and mitochondrial contamination and increased the number of prokaryotic reads for the root endosphere, leaf endosphere, and phyllosphere samples respectively. The amplification reactions were conducted in 96-well plate format, and for each sample were performed in triplicate with ~10ng template per reaction. The PCR conditions used were 94 degrees C for 3 min, followed by 30 cycles of 94 degrees for 45s, 78 degrees for 10s, 50 degrees for 60s, and 72 degrees for 90s, and finally by 72 degrees for 10 min then cooling to 4 degrees. Four negative controls and several technical replicates were included in each 96-well plate to measure levels of contamination and to determine OTU measurability thresholds (see Fig. S2). Triplicate reactions for each sample were pooled, and quantification was carried out with the Qubit High Sensitivity Assay kit (Life Technologies) on a Turner Biosystems fluorescence plate reader (Promega, Madison, WI, USA). Sets of 96 barcoded PCR products were pooled in equimolar ratios and cleaned up using the AMPureXP magnetic beads (Beckman-Coulter, Indianapolis, IN, USA). Paired-end 2 x 250bp sequencing of the barcoded amplicons was performed on a MiSeq machine running v2 chemistry (Illumina Inc, San Diego, CA, USA).

**Diversity analyses**

We calculated the Shannon Diversity (H ́) using the package “BiodiversityR” in R. These values were calculated from the taxonomic abundance matrices generated from all measurable, rarefied OTUs.

**Exozymes analysis through flourogenic substrates**

A detailed method with some modifications described by Khan et al. (2016) was used for the quantification of phosphatase, cellulase, glucosidase, and esterase. The rhizospheric soil samples from all plants were incubated in sodium acetate buffer (pH 5.2) for 24 hrs on shaking (150rpm) and the supernatants were harvested using centrifugation (4°C, 12,000 rpm for 15 min). The filtrates (F) were syringe filtered (0.22 μm) to remove traces of turbidity. Three different types of exozymes phosphatase, glucosidases and cellulase were quantified on Florescence spectrophotometer. For each type of enzyme analysis, a minimum of three replicates for each substrate (F + buffer + substrate), a quenched standard (sample + buffer + 4-MUB), and a substrate control (buffer + substrate) were maintained. The total volume of liquid in the cuvette was 2 mL F or buffer and 100 µL substrate or 4-MUB with different types of F obtained from soils. The pre-optimized fluorescence spectrophotometer (Shimadzo, Tokyo, Japan) was used to read the absorbance with 360 nm excitation and 460 nm emission at time zero and 30-minute intervals for 2 hours. The readings were calculated according to this formula:

Activity (μmol h-1 L-1) = slope of concentration versus time in hours

Concentration = Raw Activity × 2.5 = (Assay – Substrate Control) / {[(Standard – Water) / (10 μmol/L × 0.00005 L)] × 0.0002 L}

**Indole acetic acid analysis**

The estimation of the level of indole-3-acetic acid (IAA) in the culture broth was performed using colorimetric assay as shown by Hoffman et al. [1]. All the isolated endophytes were cultured in 20 mL Czapek broth with and without L-tryptophan for seven days (incubated at 30 ± 2°C; 200 rpm). The cell-free cultures obtained after centrifugation (10,000 × *g* for 10 min at 4°C) were filtered through a 0.45-µm cellulose acetate filter (DISMIC®, Denmark). 1 N HCl was used to acidify the cell-free cultures (pH 2.8) and subsequently extracted 3 times with 20 mL ethyl acetate. The extracted fractions were combined prior to evaporate under a vacuum at 45°C in a rotary evaporator. The resultant residue was re-suspended in 3 mL 50% methanol: water and one mL of it was mixed with 2 mL Salkowski reagent (12 g FeCl_3_/L of 7.9 M H_2_SO_4_) for one hour in dark condition. Readings for change in color was noted at 535 nm in ELISA Spectrophotometer (xMark BioRad, USA). The IAA in culture broth was quantified against separately prepared standard IAA (Sigma-Aldrich, Korea). A total of five replications (100 ml in Erlenmeyer flask) were used to make sure the validation of IAA results.

After finding positive results for endophytes, we selected one of the bioactive strains that showed maximum IAA production in the colorimetric assay for further quantification with UPLC as described in the method of Khan et al. [34] (UPLC-ESI-MS/MS conditions mentioned in Table SII). Two external standard preparations (IAA dissolved in 100% water) and their average response factor was used for the quantification of IAA. Therefore the area under the peak for each Multiple Reaction Monitoring (MRM) trace was integrated and obtained by the transition of the precursor ion (175.65) to the product ion (129.8) for both the standards and the samples. The purity of standard (98% w/w) was also considered to calculate response factor.

**References**

1. Hoffman, M.T., Gunatilaka, M.K, Wijeratne, K., Gunatilaka, L., Arnold, A.E. (2013). Endohyphal bacterium enhances production of indole-3-acetic acid by a foliar fungal endophyte. PlosOne, 8(9):e73132.
